# HIV pre-exposure prophylaxis and diagnoses of sexually transmitted infections – observational data from German checkpoints, 01/2019–08/2021

**DOI:** 10.1186/s12889-023-15570-6

**Published:** 2023-04-07

**Authors:** Ulrich Marcus, Susanne B. Schink, Christoph Weber

**Affiliations:** 1 Department for Infectious Diseases Epidemiology, Robert Koch-Institute, Berlin, Germany; 2 Checkpoint BLN, Berlin, Germany

**Keywords:** HIV pre-exposure prophylaxis, PrEP, Men who have sex with men, Sexually transmitted infections, Germany

## Abstract

**Background:**

The impact of starting HIV pre-exposure prophylaxis (PrEP) on diagnoses of sexually transmitted infections (STI) remains unclear. We used data from German HIV/STI Checkpoints collected from 01/2019 to 08/2021 to determine the impact of PrEP use on syphilis, gonorrhoea and chlamydia diagnoses.

**Methods:**

We used self-reported data on demographics, sexual behaviour, testing and PrEP use, as well as lab-confirmed diagnoses from visits to HIV/STI Checkpoints in Germany. PrEP use was categorized as (1) never used; (2) intention to use; (3) former use; (4) current on-demand use; (5) daily use. In multivariate regression analyses (MRA) with gonorrhoea, chlamydia, and syphilis diagnoses as outcomes, we controlled for age, number of sexual partners, number of condomless anal intercourse (CAI) partners in the last six months, and testing recency.

**Results:**

For the analysis, we included 9,219 visits for gonorrhoea and chlamydia testing and 11,199 visits for syphilis testing conducted at checkpoints from 01/2019 to 08/2021. MRA identified age (aOR 0.98; 95%CI 0.97–0.99), number of sexual partners in the past six months (aOR 4.90; 95%CI 2.53–9.52 for 11 + partners), and use of chemsex substances (aOR 1.62; 95%CI 1.32-2.00) as risk factors for gonorrhoea, while age (aOR 0.99; 95%CI 0.98-1.00), number of CAI partners (aOR 3.19; 95%CI 2.32–4.41 for 5 + partners), partner sorting (aOR 1.30; 95%CI 1.09–1.54), and use of chemsex substances (aOR 1.29; 95%CI 1.05–1.59) were risk factors for chlamydia infections. For syphilis, the number of CAI partners (aOR 3.19; 95%CI 1.60–6.34 for 5 + partners) was found to be the only significant risk factor. There was a strong association between PrEP use and the number of sexual partners (≤ 5 vs.>5: aOR 3.58; 95%CI 2.15–5.97 for daily PrEP use), the number of CAI partners in the past six months (≤ 1 vs.>1: aOR 3.70; 95%CI 2.15–6.37 for daily PrEP use), and the number of STI tests performed (suggesting higher testing frequency). Both outcomes were also related to partner sorting, chemsex, and selling sex.

**Conclusions:**

Checkpoint visits reporting current PrEP use or intention to start PrEP correlated with eligibility criteria for PrEP, i.e. high partner numbers, inconsistent condom use during anal intercourse, and use of chemsex drugs. Use of HIV-specific prevention methods such as HIV serosorting, PrEP sorting, and viral load sorting was reported more frequently. (Daily) PrEP use was an independent risk factor for a chlamydia diagnosis only.

**Supplementary Information:**

The online version contains supplementary material available at 10.1186/s12889-023-15570-6.

## Background

There are many, partly conflicting data on the impact of starting HIV pre-exposure prophylaxis (PrEP) on the diagnosis of sexually transmitted infections (STI). Even systematic reviews arrive at different conclusions [1–6]. Reasons for the diverging results could be, e.g., differences in the baseline STI risk between populations compared, insufficient controlling for increased STI screening frequency within PrEP programmes, aggregate vs. single infection outcome measures, or neglected change in STI diagnoses rates over time.

In Germany, testing is offered by primary care physicians, specialists, and local health departments. However, barriers to testing in primary care include (1) the lack of insurance coverage for STI screening for asymptomatic infections and (2) the requirement to disclose sexual risk behaviours in order to receive HIV/STI testing covered by health insurance. To improve access to queer-friendly HIV and STI testing, Checkpoints were established and managed by non-governmental organisations in Germany. Checkpoints are low-threshold community-based testing and counselling sites [7, 8].

PrEP for HIV was officially approved in Germany in 2016, but access remained severely restricted due to prohibitively high drug costs and lack of health insurance coverage until October 2017, when a drastic price cut for a generic PrEP version greatly increased PrEP affordability [9]. Since September 2019, check-ups, medical advice, HIV/STI testing and the actual PrEP prescription are covered by the statutory health insurance system in Germany, provided the prescribing physician is licensed to prescribe PrEP [10]. Until September 2019, HIV/STI Checkpoints in Germany were a low-cost alternative for HIV and STI testing for PrEP users compared to the primary care system. After September 2019, Checkpoints remain a low-cost alternative for PrEP users with private health insurance and co-pay, for PrEP users without health insurance, and for PrEP users with access barriers to licensed PrEP prescribers, as well as for PrEP users who are insured but do not wish to disclose PrEP use to their health insurance provider.

We used observational data from HIV/STI Checkpoints collected in Germany from 01/2019 to 08/2021 to determine the association between PrEP use and the diagnosis of an infection with syphilis, gonorrhoea and/or chlamydia.

## Methods

We used biobehavioural data routinely collected at consultation visits in HIV/STI Checkpoints in Germany from 01/2019 to 08/2021. At each visit, a voluntary and anonymous questionnaire is self-administered on a tablet. Laboratory test results are subsequently added to the questionnaire, resulting in an anonymous bio-behavioural dataset of Checkpoint visits. Individual participant data cannot be matched to prior visits. Questions include demographics (month and year of birth, country of birth, country of residence, level of education, employment status, as well as gender, sexual orientation and relationship status), sexual behaviour (number, gender and type of partners in the last 6 months; condom and PrEP use), substance use when having sex and type of substances used, place and recency of previous HIV and STI tests, hepatitis vaccination status, self-assessed risk estimate for HIV infection, reasons for testing, and reasons for not using condoms. Questionnaires cannot be linked to individuals but represent the total number of consultations, i.e. a person might have filled out more than one questionnaire. The subgroup of self-identified heterosexual clients was excluded from this analysis because PrEP use was very infrequent (< 1%) in this client subgroup. Ethic board approval was granted by the Ethics Review Board of the Berlin Medical Association (Eth-61/21).

Based on answers to questions on reasons for testing and on PrEP use since the previous HIV test, we constructed a secondary variable to categorize (1) no PrEP use; (2) intention to use PrEP; (3) former PrEP use; (4) current on-demand PrEP use; and (5) daily PrEP use (see Additional File 1).

We used uni- and multivariate regression analyses with a diagnosis of gonorrhoea, chlamydia and syphilis as outcome while controlling for age, number of sexual partners in the last six months, and the number of condomless anal intercourse (CAI) partners in the last six months, and using the type of PrEP used as explanatory variable. Further variables included in the models as surrogate markers for distinct sexual networks and their potential impact were receiving money for sexual services, a combined variable that includes HIV serosorting, PrEP sorting, or viral load sorting as reasons for not using condoms named ‘partner sorting’ (see Additional File 1), and reporting the use of chemsex drugs in the context of recent sexual encounters.

The multivariate logistic regression models were constructed with stepwise forward variable selection for variables significant in univariate analysis. We included three variables (age, region of birth, partner sorting) in the syphilis model which were not significant in univariate analysis for better comparability with the gonorrhoea and chlamydia models. Missing values were included as a separate category if appropriate, except for age, which was included as a continuous variable.

In Germany, Checkpoints in general do not provide treatment but refer clients with an STI diagnosis to a physician. Most clients are aware of this fact, thus clients with symptoms would seek treatment directly. It is for this reason that clients in Checkpoints generally present without signs or symptoms of an STI. Data on symptoms are therefore not collected. Gonorrhoea and chlamydia diagnoses are based on positive nucleic acid amplification test results. Most gonorrhoea/chlamydia testing in checkpoints is conducted on pooled specimens, hence it is not possible to determine whether one or multiple sites were affected. A syphilis diagnosis was based on serological lab test results. We defined syphilis as “active” when the test results were in line with a recommendation to treat.

We also constructed two multivariate regression models to identify factors associated with the numbers of sex partners and the number of CAI partners. Both models used binarized outcomes: up to 5 partners or more than 5 different sex partners in the last 6 months and up to 1 partner or more than 1 partner with CAI in the last 6 months.

Clients filled out anonymous online questionnaires prior to their consultations on tablets provided at each Checkpoint. Laboratory test results were entered by Checkpoint staff or volunteers post hoc. We analysed data in Stata 17™ (StataCorp, College Station, Texas, USA).

## Results

The analysis of 11,228 Checkpoint visits from 01.01.2019 through 31.08.2021 showed that not using PrEP or not answering the question whether PrEP was used was with 72% the most common answer (see Additional File 1). Bearing in mind that returning clients could have provided a questionnaire at each visit, an interest in starting PrEP was disclosed on more than 10% of questionnaires while current PrEP use was reported on more than 16% of questionnaires (Table 1).

Matched with 9,239 tests results for gonorrhoea, 9,247 for chlamydia, and 11,228 for syphilis, the most prevalent infections were chlamydia and gonorrhoea with 11.2–11.8% during visits of current PrEP users, followed by visits of former PrEP users with 8.5% and 10.4% respectively. Syphilis was also more frequently diagnosed during visits of current PrEP users, with 2.7% in daily and 3.0% in on-demand use. The number of tests for gonorrhoea/chlamydia and syphilis, and the number and proportion of diagnoses are shown in Table 1.

**Table 1:** Gonorrhoea, chlamydia and syphilis diagnoses and PrEP use at Checkpoints visits*, Germany, 01/2019-08/2021

PrEP	consultations		gonorrhoea			chlamydia			syphilis		
	N	%	tested	pos.	%	tested	pos.	%	tested	pos.	%
**no use / not answered**	8,073	71.9%	6,314	352	5.6%	6,318	451	7.1%	8,073	90	1.1%
**intention**	1,187	10.6%	1,043	98	9.4%	1,043	95	9.1%	1,187	23	1.9%
**former use**	122	1.1%	106	11	10.4%	106	9	8.5%	122	1	0.8%
**on demand**	469	4.2%	442	50	11.3%	442	52	11.8%	469	14	3.0%
**daily**	1,377	12.3%	1,334	149	11.2%	1,339	157	11.7%	1,377	37	2.7%
**total**	11,228	100.0%	9,239	660	7.1%	9,247	764	8.3%	11,228	165	1.5%

*data represents consultations and not individual clients, repeated participation by clients was possible

Factors associated with the diagnosis of gonorrhoea in the multivariate model were primarily the number of *sex partners* in the last 6 months and age (2.4% risk reduction per additional year of age). Other factors independently associated with a gonorrhoea diagnosis were 5 or more CAI partners in the last 6 months or a missing response to this question, the intention to use PrEP, the use of chemsex drugs with recent sex partners, a previous STI test in the last 6 months, and the following regions of origin: European countries other than Germany, South America, Australia and New Zealand. Borderline significant (0.05 < p < 0.09) were the following factors: having had CAI with 1 to 5 partners in the last 6 months,, and any current PrEP use (see Table 2).

**Table 2:** Uni- and multivariate associations with a gonorrhoea diagnosis at Checkpoint visits^1^, Germany, 01/2019-08/2021

		gonorrhoea (N = 9,211)				(N = 9,144)			
		OR	95%CI		p	aOR	95%CI		p
PrEP status	PrEP never	ref.							
	PrEP intention	**1.74**	1.37	2.20	< 0.001	**1.32**	1.03	1.69	0.027
	former PrEP	**1.98**	1.05	3.74	0.034	1.39	0.73	2.67	0.316
	PrEP on demand	**2.10**	1.52	2.88	< 0.001	1.39*	0.97	1.97	*0.067*
	daily PrEP	**2.13**	1.74	2.60	< 0.001	1.30*	1.00	1.71	*0.051*
Age	per year	**0.98**	0.97	0.99	< 0.001	**0.98**	0.97	0.99	< 0.001
Region of birth	Germany	ref.							
	elsewhere in Europe	**1.91**	1.57	2.32	< 0.001	**1.52**	1.24	1.87	< 0.001
	Eastern Mediterranean	1.19	0.80	1.76	0.385	0.92	0.61	1.37	0.673
	Asia	1.25	0.85	1.83	0.259	0.99	0.67	1.47	0.965
	Africa	1.25	0.69	2.28	0.459	1.04	0.56	1.92	0.896
	USA, Canada	**1.46**	1.00	2.13	0.049	1.04	0.71	1.54	0.826
	Mexico, Central America	1.50	0.75	3.01	0.247	1.04	0.51	2.11	0.916
	South America	**2.85**	2.13	3.82	< 0.001	**2.04**	1.50	2.77	< 0.001
	Australia, New Zealand	**3.47**	2.06	5.84	< 0.001	**2.02**	1.18	3.48	0.011
	missing	0.98	0.54	1.78	0.958	**1.22**	0.60	2.50	0.581
Sex selling^2^	no sex sold	ref.							
	sex sold	**2.22**	1.62	3.04	< 0.001	1.15	0.82	1.61	0.427
	missing	0.63	0.32	1.24	0.179	074	0.32	1.69	0.470
Number of sex partners^2^	1	ref.							
	2	**2.25**	1.09	4.65	0.028	**2.20**	1.06	4.55	0.034
	3	**2.33**	1.16	4.68	0.018	**2.14**	1.06	4.34	0.035
	4	**3.24**	1.62	6.45	0.001	**2.75**	1.37	5.53	0.005
	5	**3.72**	1.91	7.28	< 0.001	**3.07**	1.55	6.07	0.001
	6–10	**4.94**	2.60	9.40	< 0.001	**3.71**	1.92	7.16	< 0.001
	11+	**8.41**	4.44	15.93	< 0.001	**4.90**	2.53	9.52	< 0.001
	missing	**3.08**	1.19	7.93	0.020	**3.07**	1.06	8.90	0.038
Number of condomless anal intercourse partners^2^	0	ref.							
	1	1.31	0.97	1.76	0.081	1.32*	0.98	1.80	*0.076*
	2–4	**1.74**	1.32	2.30	< 0.001	1.29*	0.96	1.73	*0.087*
	5+	**3.84**	2.92	5.04	< 0.001	**1.85**	1.35	2.55	< 0.001
	missing	**1.59**	1.17	2.15	0.003	**1.53**	1.11	2.11	0.010
Partner sorting^3^	no partner-sorting	ref.							
	partner-sorting	**1.30**	1.08	1.55	0.005	1.04	0.86	1.27	0.684
Chemdrug use^3^	no chemdrugs	ref.							
	chemdrugs	**2.61**	2.15	3.16	< 0.001	**1.62**	1.32	2.00	< 0.001
STI test recency	No previous test	ref.							
	≤ 3 months	**2.61**	1.90	3.59	< 0.001	**1.45**	1.02	2.07	*0.041*
	≤ 6 months	**2.15**	1.56	2.97	< 0.001	**1.45**	1.03	2.04	*0.032*
	> 6 months ago	1.29	0.93	1.77	0.124	1.08	0.78	1.50	0.646
	not answered	1.12	0.64	1.97	0.697	0.98	0.55	1.74	0.934
	missing	1.13	0.63	2.04	0.683	1.16	0.56	2.41	0.691
constant						**0.02**	0.01	0.05	< 0.001

Bold face: significance p < 0.05; * significance 0.05 ≤ p < 0.09; ^1^= data represents consultations and not individual clients, repeated participation by clients was possible; absolute numbers of visits included in the multivariate regression analyses for the different categories are shown in Additional Table 1; ^2^= last six months; ^3^= last sexual risk

Factors associated with the diagnosis of a chlamydia infection were primarily the number of *CAI partners* in the last 6 months and age (1.4% risk reduction per additional year of age). Other factors independently associated with a chlamydia diagnosis were having had more than 10 different sex partners in the last 6 months, daily PrEP use, reporting not to use condoms based on information about HIV serostatus, PrEP use or undetectable viral load of the partner, the use of chemsex drugs with recent sex partners, and having been born in Asia. A recent STI test in the last 3 months and a last STI test more than 6 months ago were both associated with a lower probability of a chlamydia diagnosis (see Table 3).

**Table 3:** Uni- and multivariate associations with a chlamydia diagnosis at Checkpoint visits^1^, Germany, 01/2019- 08/2021

		chlamydiosis (N = 9,219)				(N = 9,219)			
		OR	95% CI		p	aOR	95% CI		p
PrEP status	PrEP never	ref.							
	PrEP intention	**1.31**	1.04	1.65	0.024	1.03	0.81	1.32	0.782
	former PrEP	1.22	0.61	2.43	0.573	0.94	0.47	1.90	0.867
	PrEP on demand	**1.72**	1.27	2.34	0.001	1.30	0.93	1.82	0.121
	daily PrEP	**1.71**	1.41	2.08	< 0.001	**1.35**	1.05	1.75	0.021
Age	per year	**0.99**	0.98	1.00	0.006	**0.99**	0.98	1.00	0.004
Region of birth	Germany	ref.							
	elsewhere in Europe	1.19*	0.98	1.45	*0.071*	1.04	0.85	1.27	0.710
	Eastern Mediterranean	1.30	0.94	1.81	0.109	1.06	0.76	1.48	0.724
	Asia	**1.67**	1.23	2.25	0.001	**1.40**	1.03	1.92	0.033
	Africa	1.22	0.72	2.06	0.459	1.05	0.62	1.79	0.858
	USA, Canada	1.30	0.93	1.83	0.126	1.06	0.75	1.50	0.733
	Mexico, Central America	1.06	0.53	2.11	0.867	0.79	0.39	1.60	0.519
	South America	**1.40**	1.01	1.93	0.042	1.12	0.80	1.56	0.512
	Australia, New Zealand	**1.82**	1.03	3.22	0.039	1.29	0.72	2.32	0.391
	missing	0.52	0.26	1.02	0.056	0.54	0.25	1.18	0.121
Selling sex^2^	no sex sold	ref.							
	sex sold	**1.69**	1.22	2.33	0.002	1.08	0.77	1.52	0.649
	missing	0.65	0.35	1.20	0.171	0.85	041	1.78	0.668
Number of sex partners^2^	0	0.88	0.26	2.96	0.834	0.69	0.20	2.37	0.557
	1	ref.							
	2	1.03	0.64	1.67	0.900	0.96	0.59	1.57	0.881
	3	1.26	0.81	1.95	0.314	1.12	0.71	1.77	0.623
	4	1.21	0.76	1.90	0.424	1.00	0.62	1.61	0.995
	5	**1.80**	1.18	2.74	0.006	1.46*	0.95	2.27	*0.087*
	6–10	**1.70**	1.14	2.52	0.009	1.27	0.83	1.92	0.267
	11+	**2.60**	1.76	3.84	< 0.001	**1.58**	1.03	2.42	0.037
	missing	1.41	0.69	2.88	0.344	1.65	0.72	3.81	0.236
Number of condomless anal intercourse partners^2^	0	ref.							
	1	**1.74**	1.29	2.36	< 0.001	**1.64**	1.21	2.23	0.002
	2–4	**2.83**	2.15	3.73	< 0.001	**2.38**	1.79	3.17	< 0.001
	5+	**4.55**	3.43	6.02	< 0.001	**3.19**	2.32	4.41	< 0.001
	missing	**2.10**	1.55	2.85	< 0.001	**2.07**	1.50	2.86	< 0.001
Partner sorting^3^	no partner-sorting	ref.							
	partner-sorting	**1.56**	1.33	1.84	< 0.001	**1.30**	1.09	1.54	0.003
Chemdrug use^3^	no chemdrugs	ref.							
	chemdrugs	**1.79**	1.47	2.18	< 0.001	**1.29**	1.05	1.59	0.017
STI test recency	no STI test	ref.							
	≤ 3 months	1.19	0.92	1.54	0.186	**0.66**	0.49	0.90	0.009
	≤ 6 months	1.22	0.94	1.58	0.127	0.89	0.67	1.18	0.419
	> 6 months ago	0.85	0.66	1.09	0.199	**0.77**	0.59	0.99	0.045
	not answered	1.22	0.81	1.84	0.344	1.11	0.73	1.70	0.614
	missing	0.71	0.42	1.19	0.194	0.81	0.43	1.53	0.511
constant						**0.05**	0.03	0.09	< 0.001

Bold face: significance p < 0.05; * significance 0.05 ≤ p < 0.09; ^1^= data represents consultations and not individual clients, repeated participation by clients was possible; absolute numbers of visits included in the multivariate regression analyses for the different categories are shown in Additional Table 1; ^2^= last six months; ^3^= last sexual risk

Factors associated with an acute syphilis diagnosis were primarily the number of *CAI partners* in the last 6 months (see Table 4).

**Table 4:** Uni- and multivariate associations with a diagnosis of syphilis requiring treatment at Checkpoint visits^1^, Germany, 01/2019-08/2021

		syphilis (N = 11,199)				(N = 11,199)			
		OR	95% CI		p	aOR	95% CI		p
PrEP status	PrEP never	ref.							
	PrEP intention	**1.77**	1.12	2.82	0.015	1.47	0.91	2.37	0.118
	former PrEP	0.74	0.10	5.35	0.765	0.56	0.08	4.06	0.563
	PrEP on demand	**2.77**	1.56	4.90	< 0.001	1.71	0.91	3.20	*0.093*
	daily PrEP	**2.46**	1.67	3.63	< 0.001	1.30	0.78	2.18	0.312
Age	per year	1.01	1.00	1.03	0.074	1.00	0.99	1.02	0.531
Region of birth	Germany	ref.							
	elsewhere in Europe	1.10	0.73	1.66	0.651	0.99	0.65	1.51	0.949
	Eastern Mediterranean	1.12	0.56	2.24	0.749	0.97	0.48	1.98	0.934
	Asia	1.62	0.90	2.93	0.108	1.50	0.81	2.77	0.193
	Africa	0.36	0.05	2.57	0.305	0.31	0.04	2.27	0.249
	USA, Canada	1.26	0.60	2.62	0.539	1.05	0.50	2.21	0.880
	Mexico, Central America	1.73	0.54	5.56	0.355	1.47	0.45	4.80	0.525
	South America	1.41	0.73	2.74	0.306	1.15	0.58	2.27	0.697
	Australia, New Zealand	0.65	0.09	4.74	0.675	0.46	0.06	3.40	0.449
	missing	1.94	0.89	4.24	0.097	1.87	0.73	4.83	0.195
Selling sex^2^	no sex sold	ref.							
	sex sold	**2.11**	1.16	3.84	0.014	1.46	0.78	2.75	0.235
	missing	0.56	0.14	2.25	0.410	0.22	0.04	1.14	0.071
Number of sex partners^2^	0	2.38	0.49	11.63	0.283	1.60	0.31	8.18	0.571
	1	ref.							
	2	0.96	0.36	2.59	0.939	0.96	0.35	2.61	0.936
	3	0.88	0.33	2.32	0.793	0.84	0.31	2.26	0.734
	4	1.59	0.64	3.91	0.314	1.51	0.60	3.80	0.385
	5	1.21	0.49	3.01	0.682	1.04	0.41	2.67	0.929
	6–10	1.90	0.85	4.24	0.116	1.48	0.64	3.44	0.361
	11+	**3.20**	1.46	7.03	0.004	1.86	0.79	4.39	0.157
	missing	1.74	0.45	6.80	0.423	1.04	023	4.79	0.955
Number of condomless anal intercourse partners^2^	0	ref.							
	1	**2.00**	1.04	3.88	0.039	**2.14**	1.09	4.18	0.026
	2–4	**2.27**	1.20	4.29	0.012	**1.95**	1.01	3.75	0.046
	5+	**5.94**	3.22	10.98	< 0.001	**3.19**	1.60	6.34	0.001
	missing	**3.49**	1.85	6.57	< 0.001	**2.92**	1.50	5.67	0.002
Partner sorting^3^	no partner-sorting	ref.							
	partner-sorting	1.03	0.71	1.49	0.866	0.91	0.62	1.35	0.649
Chemdrug use^3^	no chemdrugs	ref.							
	chemdrugs	**1.78**	1.19	2.68	0.005	1.27	0.82	1.96	0.277
STI test recency	no STI test	ref.							
	≤ 3 months	**2.83**	1.54	5.22	0.001	1.53	0.77	3.04	0.226
	≤ 6 months	**1.90**	1.00	3.59	0.049	1.34	0.69	2.62	0.390
	> 6 months ago	1.34	0.72	2.49	0.356	1.19	0.63	2.23	0.598
	not answered	**2.38**	1.01	5.60	0.047	1.91	0.80	4.57	0.143
	missing	**3.15**	1.37	7.24	0.007	**2.87**	1.09	7.54	0.033
constant						**0.00**	0.00	0.01	< 0.001

Bold face: significance p < 0.05; * significance 0.05 ≤ p < 0.09; ^1^= data represents consultations and not individual clients, repeated participation by clients was possible; absolute numbers of visits included in the multivariate regression analyses for the different categories are shown in Additional Table 1; ^2^= last six months; ^3^= last sexual risk

The following factors were associated with higher numbers of sex partners (i.e. more than 5) in the last 6 months: ever PrEP use or the intention to use PrEP; receiving money for providing sexual services; the use of chemsex drugs; reporting not to use condoms based on information about HIV serostatus, PrEP use or undetectable viral load of the partner; a recent STI test; and having been born in the following regions: European countries other than Germany, Eastern Mediterranean, Central or South America, Australia or New Zealand. Compared to not using PrEP, taking PrEP for less than 6 months was associated with a lower probability for high partner numbers (see Table 5).

Associated with more than one CAI partners in the last 6 months were ever PrEP use or the intention to use PrEP; receiving money for providing sexual services; the use of chemsex drugs; reporting not to use condoms based on information about HIV serostatus, PrEP use or undetectable viral load of the partner; an STI test within the last 3 months; and – compared to having been born in Germany – all other regions of origin except for Australia and New Zealand. Taking PrEP for more than 12 months was associated with a higher probability of more than 1 CAI partner in the last 6 months (see Table 5).

**Table 5:** Uni- and multivariate associations with number of sex partners (binarized) and number of CAI partners (binarized) in the last six months at Checkpoint visits^1^, Germany, 01/2019-08/2021

		≤ 5 vs. > 5 sex partners in last 6 months (N = 10,563)								≤ 1 CAI partners vs. > 1 CAI partners (N = 10,621)							
		OR	95% CI		p	aOR	95% CI		p	OR	95% CI		p	aOR	95% CI		p
Age	per year	**1.02**	1.01	1.02	< 0.001	**1.01**	1.01	1.02	< 0.001	**1.02**	1.02	1.03	< 0.001	**1.02**	1.02	1.03	< 0.001
PrEP status	PrEP never	ref.															
	PrEP intention	**2.09**	1.85	2.37	< 0.001	**1.75**	1.53	2.00	< 0.001	**1.59**	1.40	1.80	< 0.001	**1.34**	1.17	1.53	< 0.001
	former PrEP	**2.11**	1.46	3.05	< 0.001	**2.75**	1.39	5.43	0.004	**2.35**	1.59	3.46	< 0.001	**2.35**	1.12	4.90	0.023
	PrEP on demand	**3.01**	2.47	3.66	< 0.001	**3.11**	1.82	5.29	< 0.001	**5.65**	4.38	7.29	< 0.001	**3.43**	1.92	6.14	< 0.001
	daily PrEP	**3.72**	3.28	4.21	< 0.001	**3.58**	2.15	5.97	< 0.001	**6.84**	5.81	8.06	< 0.001	**3.70**	2.15	6.37	< 0.001
Time on PrEP	not on PrEP	ref.															
	no longer on PrEP	**1.72**	1.15	2.56	0.008	0.55	0.27	1.14	0.109	**1.67**	1.09	2.55	0.018	0.75	0.34	1.65	0.480
	1-<6 months	**2.47**	2.05	2.98	< 0.001	**0.55**	0.32	0.93	0.026	**3.36**	2.69	4.19	< 0.001	1.02	0.58	1.81	0.942
	6–11 months	**2.95**	2.36	3.68	< 0.001	0.63	0.36	1.08	0.095	**4.82**	3.60	6.45	< 0.001	1.42	0.77	2.60	0.259
	12 + months	**3.73**	3.20	4.36	< 0.001	0.78	0.46	1.31	0.342	**7.82**	6.22	9.84	< 0.001	**2.26**	1.27	4.03	0.006
STI test recency	no STI test	ref.															
	≤ 3 months	**4.75**	4.13	5.47	< 0.001	**2.55**	2.17	2.99	< 0.001	**3.48**	3.04	3.98	< 0.001	**1.33**	1.14	1.56	< 0.001
	≤ 6 months	**3.35**	2.92	3.85	< 0.001	**2.41**	2.08	2.79	< 0.001	**1.76**	1.55	1.99	< 0.001	1.08	0.94	1.25	0.257
	> 6 months ago	**2.08**	1.83	2.36	< 0.001	**1.74**	1.53	1.99	< 0.001	**1.20**	1.07	1.34	0.002	0.97	0.86	1.10	0.671
	not answered	**1.65**	1.33	2.05	< 0.001	**1.34**	1.06	1.68	0.014	**1.75**	1.42	2.15	< 0.001	**1.39**	1.11	1.73	0.004
Region of birth	Germany	ref.															
	elsewhere in Europe	**1.32**	1.20	1.46	< 0.001	**1.23**	1.11	1.37	< 0.001	**1.25**	1.13	1.38	< 0.001	**1.22**	1.10	1.37	< 0.001
	Eastern Mediterranean	**1.30**	1.09	1.53	0.003	**1.40**	1.16	1.68	< 0.001	**1.64**	1.38	1.95	< 0.001	**1.76**	1.46	2.13	< 0.001
	Asia	0.91	0.77	1.08	0.283	0.93	0.77	1.12	0.428	**1.39**	1.17	1.64	< 0.001	**1.42**	1.18	1.72	< 0.001
	Africa	1.15	0.87	1.51	0.327	1.20	0.89	1.63	0.231	**1.73**	1.30	2.31	< 0.001	**1.99**	1.45	2.73	< 0.001
	USA, Canada	**1.52**	1.26	1.83	< 0.001	1.17	0.96	1.43	0.128	**1.44**	1.19	1.74	< 0.001	1.20	0.97	1.48	0.091
	Mexico, Central America	**1.59**	1.13	2.25	0.009	**1.54**	1.07	2.22	0.021	1.24	0.87	1.75	0.236	1.39*	0.95	2.02	0.087
	South America	**1.87**	1.56	2.23	< 0.001	**1.71**	1.41	2.07	< 0.001	**1.37**	1.15	1.65	0.001	**1.41**	1.15	1.72	0.001
	Australia, New Zealand	**2.39**	1.62	3.52	< 0.001	**1.63**	1.08	2.45	0.020	**1.91**	1.29	2.83	0.001	1.30	0.84	2.02	0.231
Selling sex^2^	no sex sold	ref.															
	sex sold	**3.05**	2.45	3.80	< 0.001	**2.87**	2.25	3.65	< 0.001	**2.96**	2.33	3.76	< 0.001	**2.62**	2.02	3.41	< 0.001
Chemdrug use^3^	no chemdrugs	ref.															
	chemdrugs	**2.54**	2.24	2.87	0.000	**2.14**	1.86	2.45	0.000	**2.68**	2.33	3.09	0.000	**2.86**	2.45	3.35	0.000
Partner sorting^3^	no partner-sorting	ref.															
	partner-sorting	**1.31**	1.20	1.43	0.000	**1.21**	1.09	1.33	0.000	**1.47**	1.34	1.62	0.000	**1.96**	1.77	2.16	0.000
constant						**0.18**	0.15	0.22	0.000					**0.29**	0.24	0.35	0.000

Bold face: significance p < 0.05; * significance 0.05 ≤ p < 0.09; ^1^= data represents consultations and not individual clients, repeated participation by clients was possible; absolute numbers of visits included in the multivariate regression analyses for the different categories are shown in Additional Table 1; ^2^= last six months; ^3^= last sexual risk

## Discussion

Our analyses confirmed that PrEP users and Checkpoint clients with the intention to use PrEP met sexual risk criteria for PrEP eligibility such as higher partner numbers, inconsistent or no condom use for anal intercourse, and chemsex substance use. They were also more likely to use HIV-specific prevention methods such as HIV serosorting, PrEP sorting, and viral load sorting.

Beyond these shared characteristics, there were noticeable differences regarding factors associated with the diagnosis of specific bacterial STIs. For a gonorrhoea diagnosis, there was an almost linear relationship with the number of sex partners and the number of CAI partners in the last six months, suggesting that not only CAI but also a range of other sexual practices contribute to the sexual transmission of gonococci [11–15]. From our data, we were unable to determine whether the number of partners increases or condom use declines after initiating PrEP. The association of both findings with the length of PrEP use would be compatible with either explanation, but might also be due to a cohort effect of earlier initiation of PrEP use by MSM with higher partner numbers and less consistent condom use. However, other observations from longitudinal data on PrEP use also suggest that condom use declines and partner numbers moderately increase [1, 5, 16–18]. Contrastingly, it appears that primarily the lack of condom use for anal intercourse contributes to an increased transmission risk of chlamydia and syphilis. This is consistent with the finding that pharyngeal chlamydia infections are rare [19], and that (painless) primary and secondary syphilis lesions remain more often undetected in the rectum than in the mouth or on the genitals.

The strong association in univariate analysis of STI diagnoses with current PrEP use and intention to use PrEP is losing significance in multivariate analysis and could be credited to higher numbers of sexual partners, respectively CAI partners. The remaining associations might be explained by sexual network effects or by testing effects. Persons taking PrEP for example may form sexual networks with other persons taking PrEP or being successfully treated for HIV. The association of shorter testing recency with higher probability of a gonorrhoea diagnosis might either be related to the association between shorter testing intervals with higher partner numbers or it may represent a paradoxical effect of frequent screening and treatment of asymptomatic infections. The association of the shorter testing interval with partner numbers has been controlled for in our analysis. The paradoxical association of frequent screening and treatment of asymptomatic infections with more self-reported bacterial STIs might be due to the arrested immunity hypothesis, i.e. a reduction in lasting immunity due to treatment leading to an increased susceptibility for reinfection [20]. This association has also been observed in a sample comparing prevalence in European MSM between 2010 and 2017 [21].

In general, we would expect a stronger association between testing frequency and diagnosis of gonorrhoea and chlamydia, but the association cannot be confirmed because we can only look at data from visits and not from individual client histories and cannot discern differences in STI testing frequencies.

The different patterns of STI infections by region of origin are not readily accounted for by the numbers of sex partner or the number of CAI partners, and point to the need for more research into migration-associated vulnerabilities and behavioural diversity of migrants from different regions.

### Limitations

Due to a lack of a unique identifier, we cannot identify individuals and their repeat visits. People at higher risk for an STI might be over-represented because they are more likely to be on PrEP and/or visit Checkpoints more frequently. Overall, 73% of daily PrEP users have been tested for STIs within the last 3 months while only 11% of non-PrEP users have done so. Nevertheless, we can compare the risk of having acquired an STI since the last STI testing between PrEP users and non-PrEP users. Untreated asymptomatic gonorrhoea usually clears within 3 months; infections would therefore not accumulate with longer testing intervals, and very few, if any checkpoint clients are testing more frequently than every three months. Thus, our comparison between PrEP users and non-PrEP users regarding the risk of acquisition of gonorrhoea may not be substantially biased by higher testing frequencies. Compared to gonorrhoea, infections with chlamydia take longer to clear and thus chlamydia cases may accumulate with longer testing intervals [22–25]. This may increase the probability to detect chlamydia among checkpoint clients with longer testing intervals, thus this may weaken the association with PrEP use. As for syphilis, a diagnosis is usually based on the detection of long-lasting antibodies. Titres decline after successful treatment and are boosted by re-infection. Compared to non-PrEP users, testing PrEP users more frequently for syphilis would result in earlier detection and treatment. In the absence of a documented treatment history, it may be difficult to discern whether an infection is new and untreated or a successfully treated re-infection because both can show elevated titres. Thus, for syphilis the impact of more frequent checkpoint visits on associations in the MRA is the least predictable among the three STIs. To summarize, the prevalence of infections among checkpoint clients is biased by different testing intervals, which should be considered when interpreting the associations found with PrEP use.

Regarding chlamydia testing, there was no subtyping conducted to identify serotypes L1-L3. Due to a relatively high prevalence of lymphogranuloma venereum infections (LGV) among MSM from Germany diagnosed with HIV [26] and reduced HIV serosorting among PrEP users, the prevalence of LGV among PrEP users in Germany may be currently increasing.

## Conclusion

Our analysis suggests that the higher STI prevalence detected during visits by PrEP users compared to non-users is due to (1) more frequent screening in general, (2) higher partner numbers in the case of gonorrhoea and (3) more condomless anal intercourse in the case of chlamydia and syphilis. Initiating PrEP might increase the acquisition of these STIs should PrEP users have sex with more partners and/or reduce condom use for anal intercourse. Since our data are cross-sectional, we are unable to discern this further.

## Electronic supplementary material

Below is the link to the electronic supplementary material.

**Additional File 1**: Variables based on questionnaire items (detailed description of PrEP use and partner sorting variables)

**Additional Table 1**: Absolute numbers for checkpoint visits from 01/2019 to 08/2021 for the categories and sub-categories included in multivariate regression analyses

## Data Availability

The dataset used for this analysis has been obtained from the German Checkpoint Collaborative Group (GCCG) under a data transfer agreement that prohibits sharing the dataset publicly. The dataset or aggregated data could be made available from the corresponding author on reasonable request.
