# Supplementary material for: HIV pre-exposure prophylaxis and diagnoses of sexually transmitted infections – observational data from German checkpoints, 01/2019–08/2021
Source: BMC Public Health. 2023 Apr 7;23:661. doi: 10.1186/s12889-023-15570-6 (PMC10082478; doi:10.1186/s12889-023-15570-6)
Supplement: Supplementary file 1 — Additional File 1: Variables based on questionnaire items (detailed description of PrEP use and partner sorting variables) [file 12889_2023_15570_MOESM1_ESM.pdf]

Additional File 1: Variables based on questionnaire items

Supplement to: Marcus U, Schink SB, Weber C. HIV Pre-Exposure Prophylaxis and Diagnoses of Sexually Transmitted Infections – Observational Data from German Checkpoints, 01/2019 – 08/2021. BMC Public Health 2023

PREP USE VARIABLE

__Questionnaire__

[Q1] ***"For which reason would you like to be tested for HIV and/or other sexually transmitted infections?"*** *(multiple response options)*

[Q1a] "I want to start PrEP"
…

[Q2] ***"Have you taken PrEP since your last negative HIV test?"***

[Q2a] "Yes"
If [Q2a] –> follow-up question [Q3]

[Q3] ***"How do you take PrEP?"***

[Q3a] "daily"
[Q3b] "on demand"
[Q3c] "I don't take PrEP anymore"

__"PrEP use" variable in the analysis__

1) daily PrEP use [Q3a]
2) current on-demand PrEP use [Q3b]
3) former PrEP use [Q3c]
4) intention to use PrEP [Q1a]
5) no PrEP use [none of the above]

PARTNER SORTING VARIABLE

__Questionnaire__

[Q4] *(referring to the last sexual risk situation:) "Why did you have sex without condoms?" (multiple responses options)*

[Q4a] "My partner told me that he/she is HIV negative"
[Q4b] "My partner told me that he/she takes drugs against HIV and is not infectious anymore"
[Q4c] "My partner told me that he/she takes PrEP"

__"Partner sorting" variable in the analysis__

HIV/VL/PrEP partner sorting if [Q4a] or [Q4b] or [Q4c]
