# Supplementary material for: HIV pre-exposure prophylaxis and diagnoses of sexually transmitted infections – observational data from German checkpoints, 01/2019–08/2021
Source: BMC Public Health. 2023 Apr 7;23:661. doi: 10.1186/s12889-023-15570-6 (PMC10082478; doi:10.1186/s12889-023-15570-6)
Supplement: Supplementary file 2 — Additional Table 1: Absolute numbers for checkpoint visits from 01/2019 to 08/2021 for the categories and sub-categories included in multivariate regression analyses [file 12889_2023_15570_MOESM2_ESM.pdf]

Additional Table 1

Additional Table 1: Absolute numbers for checkpoint visits[1] from 01/2019 through 08/2021 for the categories and sub-categories included in multivariate regression analyses.

		Gonorrhoea		Chlamydiosis		Syphilis	
		negative	positive	negative	positive	negative	positive
**PrEP status**	PrEP never	5,962	352	5,867	451	7,983	90
	PrEP intention	934	96	935	94	1,150	23
	former PrEP	94	11	96	9	120	1
	PrEP on demand	388	48	385	51	449	14
	daily PrEP	1,178	148	1,176	155	1,332	37
**Region of birth**	Germany	4,634	264	4,535	363	5,988	81
	elsewhere in Europe	1,674	182	1,694	162	2,151	32
	Eastern Mediterranean	443	30	431	45	594	9
	Asia	436	31	412	55	592	13
	Africa	168	12	164	16	208	1
	USA, Canada	397	33	393	41	470	8
	Mexico, Central America	105	9	106	9	128	3
	South America	394	64	411	46	523	10
	Australia, New Zealand	91	18	96	14	113	1
	missing	214	12	217	9	267	7
**Selling sex[2]**	no sex sold	8,065	597	7,966	704	10,395	151
	sex sold	298	49	302	45	391	12
	missing	193	9	191	11	248	2
**Number of sex partners[2]**	<2	648	10	626	33	925	9
	2	773	30	763	42	1,104	9
	3	1,024	41	1,000	67	1,344	10
	4	862	48	855	55	1,114	15
	5	1,092	70	1,062	102	1,365	14
	6-10	2,210	188	2,198	199	2,730	44
	11+	1,796	260	1,809	251	2,249	61
	missing	151	8	146	11	203	3
**Number of condomless anal intercourse partners[2]**	0	1,774	77	1,784	69	2,308	13
	1	1,869	106	1,853	125	2,481	28
	2-4	2,205	167	2,139	234	2,818	36
	5+	1,212	202	1,206	212	1,494	50
	missing	1,496	103	1,477	120	1,933	38
**Partner sorting[3]**	no partner-sorting	6,679	480	6,631	531	8,620	128
	partner-sorting	1,877	175	1,828	229	2,414	37
**Chemdrug use[3]**	no chemdrugs	7,640	499	7,526	622	9,856	136
	chemdrugs	916	156	933	138	1,178	29
**STI test recency**	no STI test	1,112	50	1,068	93	1,578	13
	≤3 months	1,787	210	1,812	188	2,228	52
	≤ 6 months	1,859	180	1,841	196	2,304	36
	> 6 months ago	3,165	183	3,126	231	4,080	45
	not answered	338	17	320	34	459	9
	missing	295	15	292	18	385	10

[1]= data represents consultations and not individual clients, repeated participation by clients was possible; [2]= last six months; [3]= last sexual risk

Time on PrEP

PrEP Use	N
not on PrEP	8,765
no longer on PrEP	94
1 - < 6 months	490
6 - 11 months	363
12+ months	851

Additional Table 1

Supplement to: Marcus U, Schink SB, Weber C. HIV Pre-Exposure Prophylaxis and Diagnoses of Sexually Transmitted Infections – Observational Data from German Checkpoints, 01/2019 – 08/2021. BMC Public Health 2023
